# The Effect of Topical Aminophylline on Hyposmia and Anosmia

**DOI:** 10.22038/IJORL.2022.64064.3195

**Published:** 2022-07

**Authors:** Hesam Jahandideh, Pardis Rahimi, Pegah Foroughi Mobarakeh, Fatemeh Dehghani Firouzabadi, Maryam Roomiani, Mohammad Farhadi

**Affiliations:** 1 *Department of Otolaryngology-Head and Neck Surgery, School of Medicine, Iran University of Medical Science, Tehran,, Iran.*

**Keywords:** Aminophylline, Anosmia, Hyposmia, Theophylline, Smell identification test

## Abstract

**Introduction:**

Olfactory training is accounted as a significantly beneficial therapy for hyposmia or anosmia. There is some evidence about methylxanthine usage for this issue. In the present study, we have investigated the effects of topical aminophylline in hyposmic and anosmic patients.

**Materials and Methods:**

In this clinical trial study, patients were randomly divided into two groups (n= 20/each), the case group was given aminophylline drops over a three-month period (using the contents of the vial aminophylline in the form of nasal drops, 250 micrograms daily) with olfactory training and the control group was given normal saline drops with olfactory training over a three-month period. The olfactory capacities were assessed before the start and after the completion of treatments using a valid and reliable smell identification test.

**Results:**

In the saline and aminophylline groups, the mean ± SD relative changes in SIT score were 0.55±0.31 and 0.85±0.56, respectively. As a result, the SIT score in the saline group climbed by 55 percent but increased by 85 percent in the aminophylline group. The difference in SIT score between pre- and post-test was meaningful in both groups (P< 0.001). The aminophylline group scored significantly higher according to the marginal longitudinal regression model, adjusting baseline parameters.

**Conclusions:**

Intranasal aminophylline plus olfactory training significantly improved SIT scores in severe hyposmia or anosmia. Hypothetically, these effects are mediated through changes in cAMP and cGMP.

## Introduction

Olfactory disorders include a wide range of dysfunctions diagnosed in many conditions such as smoking, air pollution, Parkinson's disease, Alzheimer's disease, viral infections, and traumatic brain injury (1,2). Patients can experience varying degrees of hyposmia, dysosmia, or anosmia, which can be unilateral or bilateral (1). 

It has been reported that 75% of people over the age of 80 and 50% of people between the ages of 60 and 80 experience some degrees of olfactory disorders (3,4). It suggests that older people have a poor understanding of their olfactory function. According to studies, the prevalence of olfactory disorders in the general population of communities is about 1-3%, and of course, may affect a higher percentage of people in the community (up to 16%) depending on the situation (5). Since 2019, with the onset of the Covid-19 pandemic, the prevalence of different Covid-19 related consequences like olfactory disorders, and commonly anosmia and hyposmia has increased rapidly (6-8). 

This disorder has reduced the quality of life, and various therapists have sought to treat it and restore the sense of smell in patients. As we all know, neurons associated with the olfactory nerve are one of the areas where neurogenesis does not stop after birth, and this is a window of hope for therapists to treat olfactory dysfunction with various therapeutic strategies (9,10). Furthermore, there has been previous evidence emphasizing the effectiveness of olfactory training in patients with hyposmia or anosmia, and the potency of this method has been well established (11).

So far, many studies have been conducted to find the molecular aspects associated with olfactory disorders. Some investigations have shown that cyclic adenosine monophosphate (cAMP) and cyclic guanosine monophosphate (cGMP) in olfactory mucosa are lower in hyposmic and anosmic patients than in normal individuals (12,13), so phosphodiesterase inhibitors such as theophylline can be used to increase cAMP and cGMP of the olfactory mucosa (14,15). 

Aminophylline is one of the drugs in the methylxanthine class that relaxes the muscles of the respiratory wall by blocking the enzyme phosphodiesterase and the antagonistic effect of the adenosine receptor (16). 

This drug is a combination of theophylline and ethylenediamine, by adding the ethylenediamine improves the solubility and faster impact of aminophylline than theophylline (16). Therefore, due to the importance of appropriate treatment methods and the lack of comprehensive and sufficient studies on this issue, we decided to investigate the effects of adding topical aminophylline to olfactory training in hyposmic and anosmic patients referred to the otolaryngology clinic of Iran University of Medical Sciences hospital.

## Materials and Methods

*Study population*

The present clinical trial study was approved by the Department of Otolaryngology, School of Medicine; Iran University of Medical Sciences (*IUMS*), Tehran, Iran. The study aimed to evaluate the effects of adding topical aminophylline to olfactory training in hyposmic and anosmic patients referred to the otolaryngology clinic. Also, our study was approved by the ethics committee of the *IUMS* (No.1398.477) under the registration number of IR.IUMS.FMD.REC.1398.477. Also, the study was recorded in the Iranian Registry of Clinical Trials (IRCT20220104053625N1). Accordingly, informed consent was obtained from all the patients. All study protocols were followed by the Tenets of the Declaration of Helsinki. All patients entered the study with satisfaction and knowledge by providing explanations to them and their families.

This clinical trial was performed from February 2020 to February 2021 in Firoozgar Hospital, affiliated with *IUMS*, Tehran, Iran. Firstly, demographic information was entered into a form. 

The inclusion criteria for patients to enter the trial study were: age older than 18 years, diagnosis of severe hyposmia or anosmia, duration of olfactory dysfunction was more than six months, and signing the written informed consent to participate in this study. Exclusion criteria were: lack of proper compliance to the prescribed drugs, history of nasal or skull base surgeries, history of nasal polyps, history of facial or nasal trauma, allergies to aminophylline, diagnosis of the disease less than six months before the study, consumption of other drugs.

***Study interventions***

Patients were randomly divided into two groups (n= 20/each). Due to the fact that the present investigation was a pilot and according to a methodological study by Hertzog in the field of pilot studies (17), 10 to 40 patients based on the clinician viewpoint can be assigned to each group, and we allocated 20 patients in each group.

The case group was given aminophylline drops over a three-month period (using the contents of the vial aminophylline in the form of nasal drops, 250 micrograms daily) with olfactory training. Aminophylline 250 mg ampoule was diluted with normal saline serum, as each milliliter of solution contained 250 micrograms of aminophylline, and four drops were used every 12 hours on each side of the nose in the Mygind position holding each side for 30 seconds. The control group was given normal saline drops with olfactory training over a three-month period. To double-blind the study, the nurse was unaware of the group codes. The two groups were scheduled for olfactory training at the two-time points of morning and night. The drops were instilled every 12 hours, two hours after olfactory training for both groups.

***Follow-up procedure and data extraction***

Olfactory training was done using four reagents with different odors, including rose, eucalyptus, lemon, and cloves. Each odorant was smelled for 10s/time, and the interval between two odorants was 10s. The training frequency was one time before breakfast and one time before sleep every day for three months. The olfactory capacity was assessed before the start of treatment as well as after the completion of therapy using the smell identification test/SIT. The SIT validity and reliability have already been confirmed (18,19). The SIT tab contains 24 scents, which people are asked to scroll to select the closest option that describes that scent. Based on the results of this test, patients were divided into four groups according to the scoring: normal (19-24), mild hyposmia (14-18), severe hyposmia (10-13), and anosmia (0-9). Trigeminal nerve stimuli and malingering were also diagnosed. Data on olfactory capacity before and after interventions were gathered and compared between groups.

***Statistical analysis***

Qualitative data were reported as percentage and frequency, and quantitative data as mean ± SD. Chi^2^ or Fisher’s exact test was used to examine the relationship between qualitative variables. Independent T-test or Mann-Whitney U test was used to compare variables between groups. The difference in the change in SIT score between the intervention and control groups was ultimately assessed by the marginal longitudinal regression modeling adjusted for time (pre- and post-test) and body mass index (BMI). Statistical analysis was performed using SPSS statistical software, version 19.0 (SPSS Inc., Chicago, IL, USA). A P-value <0.05 was considered a statistically meaningful difference.

## Results

**Fig 1:**
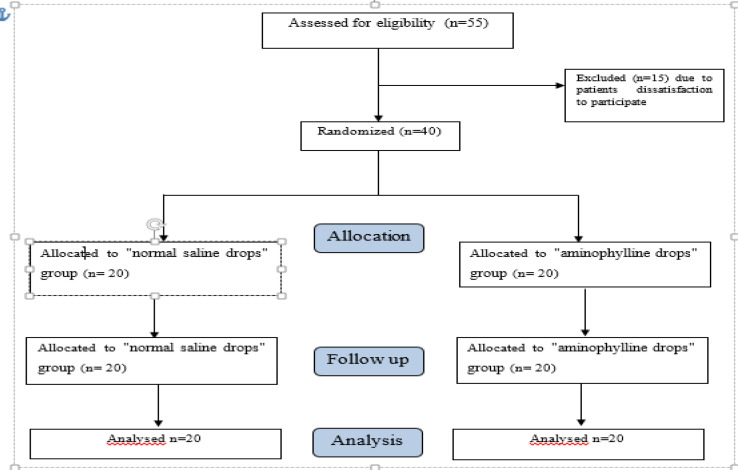
The CONSORT diagram of the study

Initially, 15 out of identified 55 patients were excluded due to lack of meeting inclusion criteria, declining participation, or other reasons. Therefore, 40 patients were randomly allocated into intervention and control groups, and thus, 40 patients (20 patients in each group) were included in the final assessment (Figure 1). The results of statistical tests showed that saline and aminophylline group patients were homogenous based on age, gender, smoker, sinusitis, respiratory viral infection, diabetes mellitus, Parkinson’s disease, Alzheimer’s disease, facial trauma, facial surgery, covid-19, disease duration, and pre-SIT Score variables (P> 0.05). Although, the BMI in the Saline group was significantly higher than the aminophylline group (P=0.014) (Table 1).The mean ± SD pre-SIT score of the saline patients was 8.60 ±1.81. Seventy-five percent were anosmic, and 25% were at the severe hyposmic stage. The pre-SIT score of the aminophylline group was also 8.45 ±2.1, including 65% anosmic and 35% severe hyposmic grades. The post-SIT scores of the saline and aminophylline patients were 12.67±1.30 and 15.21 ±3.35, respectively. There was no significant difference in pre-SIT scores between the two groups (Independent T-test: P=0.815). Furthermore, in the saline and aminophylline groups, the mean± SD relative changes in SIT score were 0.55 ±0.31 and 0.85±0.56, respectively. As a result, the SIT score in the saline group climbed by 55 percent, but it increased by 85 percent in the aminophylline group (Tables 2 and 3).

**Table 1:** Basic and demographic characteristics of the patients

**Variables**	**Saline Group** **(n=20)**	**Aminophylline Group** **(n=20)**	**P-value**
**Quantitative**	**Mean±SD**	**Mean±SD**	
Age	44.18±14.55	51.58±14.06	0.110 ^t^
BMI	27.91±3.90	25.13±5.04	0.014 ^m^
Disease Duration	5.58±2.11	9.25±10.25	0.211 ^m^
Pre-SIT Score	8.60±1.81	8.45±2.1	0.815 ^t^
Qualitative	Frequency (Percentage)	Frequency (Percentage)	P-value
Gender (male)	8 (40.0)	14 (70.0)	0.057 ^c^
Smoker	9 (45.0)	10 (50.0)	0.752 ^e^
Sinusitis	0 (0.0)	0 (0.0)	-
Covid-19 Infection	7 (35.0)	7 (35.0)	1.000 ^c^
Respiratory Viral Infection	8 (40.0)	8 (40.0)	1.000 ^c^
Diabetes Mellitus	9 (45.0)	9 (45.0)	1.000 ^c^
Parkinson’s Disease	0 (0.0)	0 (0.0)	-
Alzheimer’s Disease	0 (0.0)	2 (10.0)	0.487 ^e^
Facial Trauma	0 (0.0)	0 (0.0)	-
Facial Surgery	0 (0.0)	0 (0.0)	-

^t^: Independent T-test;^ m^: Mann-Whitney U test; ^e^: Exact Fisher’s test; ^c^: Chi-square test.

**Table 2:** Mean ± SD Pre, Post and Relative change of SIT score in Saline and Aminophylline groups

**Variable**	**Saline Group** **(n=20)**	**Aminophylline Group** **(n=20)**
Pre-SIT Score	8.60±1.81	8.45±2.1
Post-SIT Score	12.90 (1.16)	14.80 (2.90)
Relative Change in SIT score*	0.55 (0.31)	0.85 (0.56)

*: Computed as

**Table 3:** Frequency of smell test state before and after intervention

**Smell State**	**Saline Group**		**Aminophylline Group**	
	**Before Intervention**	**After Intervention**	**Before Intervention**	**After Intervention**
Anosmia	15 (75.0)	2 (10.0)	13 (65.0)	1 (5.0)
Severe Microsomia	5 (25.0)	12 (60.0)	7 (35.0)	7 (35.0)
Moderate Microsomia	0 (0.0)	6 (30.0)	0 (0.0)	7 (35.0)
Normosmia	0 (0.0)	0 (0.0)	0 (0.0)	5 (25.0)
				

The marginal longitudinal model's results are shown in Table 4. This model was used to compare pre- and post-test SIT scores across two groups while controlling for the effect of the BMI variable. The interaction effect between group and time was significant (P=0.006). In post-time, the aminophylline group's average SIT was 1.82 points higher than the saline group's. The mean difference between the SIT pre- and post-test in the aminophylline group was 6.35. In the saline group, the average difference between the SIT pre- and post-test was 4.30. All of these differences were noteworthy. 

The difference in SIT score between pre- and post-test was meaningful in both groups, with a significantly higher score in the aminophylline group.

**Table 4:** Results of marginal longitudinal data to compare SIT score in two groups at pre- and post-test

**Variable**	**Category**	**Coefficient regression**	**Standard Error**	**P-value**
Group	Aminophylline	-0.13	0.56	< 0.001
Time	Post	4.30	0.38	< 0.001
Time*Group	Aminophylline* Post	2.05	0.74	0.006
BMI	-	0.01	0.05	0.876
				

## Discussion

The present study evaluated the effect of aminophylline drops on olfactory function in patients with anosmia and hyposmia. Based on the literature review, this study was one of the first investigations in this field. According to our results, the administration of aminophylline improved subjects with anosmia and progression to hyposmia, as the number of recovered patients was more in the case group than in the controls. Of course, it is noteworthy that the improvement of the sense of smell was also seen in the placebo group, but this improvement was more in the patients who received aminophylline. It is not possible to say definitively how aminophylline increases the recovery of anosmia, but the most likely cause can be traced to possible alterations in cAMP. Aminophylline, as a combination drug of theophylline and ethylenediamine, can increase cAMP in the olfactory mucosa. Increased cAMP plays a crucial role in sodium influx and creating action potential in olfactory neurons (20). However, since our study did not determine alterations of cAMP, a definitive statement on this subject is impossible, and the issue is open to discussion. Previous studies have been carried out on the effectiveness of various therapies in improving the olfactory function in patients. To date, several studies have investigated the effect of theophylline on olfactory improvement in patients with olfactory disorders (21, 22). In this study, we used aminophylline, which appears to be more effective (due to its combination with ethylenediamine). In 2012, Henkin et al., in a pilot study, examined the effect of intranasal theophylline drops in patients with hyposmia and hypogeusia (23). They simultaneously compared intraoral theophyllinewith the results. They showed that intranasal theophylline drop performed better and was safer than its intraoral. Consistent with the results of Henkin et al., our results indicated that the intranasal drop of aminophylline, of which theophylline is a major component, had a positive outcome in the recovery of hyposmia patients. In a new study, Hosein et al. (2022) showed that intranasal theophylline drops improve olfactory function in patients with hyposmia by altering interleukin-10 and nuclear factor kappa-B in the nasal mucosa, which was in agreement with our results (24). The present study found that olfactory training is a very effective method in improving the sense of smell in patients with hyposmia and anosmia. Due to the fact that during the corona pandemic, different symptoms, especially loss of olfactory sense, are seen in many patients with Covid-19, olfactory training, as an available technique, is important in restoring the sense of smell (25-27). The effectiveness of olfactory training has been reported in many other studies. Qiao et al. (2019) investigated the effect of olfactory training on confirmed cases of upper respiratory tract infection-induced olfactory dysfunction (28). They reported that the mentioned technique improved the sense of smell in 41 percent of patients after six months. In addition, Endiyarti et al. (2018) achieved similar results, except that they examined hyposmia in patients with aging (29). In a recent study, Mahmut et al. (2020) stated that olfactory training improves the sense of smell by increasing the volume of the olfactory bulb (30), the location of the synapse of the first and second olfactory neurons. Our results were also in line with these findings showing the effectiveness of olfactory training on olfactory functions.As we know, the evaluating method of an organ function is one of the main components of a clinical trial. So far, many tests have been performed to evaluate olfactory function. In the present study, we used the Persian SIT to evaluate olfactory function in patients under consideration. The validity and reliability of the above test were confirmed by its manufacturers in 2020, and it was introduced as a valid tool for olfactory examination (18).

In our study, there were a number of patients with COVID-19 that the course of olfactory recovery in these patients is still unknown; so, in the study, we included patients who still had a loss of smell after 6 months. In routine medicine, there is generally no complete improvement in anosmia and COVID-19 may have been a justification for improving the sense of smell in 5 patients. We also had some limitations in conducting the present study. One of the limitations of the present study was the small number of samples, which confronted the results with an unwanted bias. Another limitation was the lack of evaluation of molecular studies related to the effect of aminophylline. It is better to do a similar study with larger sample size and to examine the changes in the expression of epigenetic and molecular factors in the nasal mucosa following the administration of aminophylline drops.

## Conclusion

The present study showed that treatments with intranasal aminophylline are associated with significantly improved SIT scores in patients with severe hyposmia or anosmia. We also showed that olfactory training also led to improvements in SIT scores in the control group. We suggest that the addition of intranasal aminophylline to olfactory training could be considered a safe and effective therapeutic option for patients with severe hyposmia or anosmia.
